# FoxM1 Is Associated with Poor Prognosis of Non-Small Cell Lung Cancer Patients through Promoting Tumor Metastasis

**DOI:** 10.1371/journal.pone.0059412

**Published:** 2013-03-25

**Authors:** Nuo Xu, Deshui Jia, Wenfeng Chen, Hao Wang, Fanglei Liu, Haiyan Ge, Xiaodan Zhu, Yuanlin Song, Xin Zhang, David Zhang, Di Ge, Chunxue Bai

**Affiliations:** 1 Department of Pulmonary Medicine, Zhongshan Hospital, Fudan University, Shanghai, China; 2 Shanghai Medical School, Fudan University, Shanghai, China; 3 Department of Biostatistics, School of Public Health, Fudan University, Shanghai, China; 4 Department of thoracology, Zhongshan Hospital, Fudan University, Shanghai, China; 5 Molecular Pathology Division, Department of Pathology, Mount Sinai School of Medicine, New York, New York, United States of America; Univesity of Texas Southwestern Medical Center at Dallas, United States of America

## Abstract

**Background:**

FoxM1 has been reported to be important in initiation and progression of various tumors. However, whether FoxM1 has any indication for prognosis in non-small cell lung cancer patients remains unclear.

**Methodology/Principal Findings:**

In this study, FoxM1 expression in tumor cells was examined first by immunohistochemistry in 175 NSCLC specimens, the result of which showed that FoxM1 overexpression was significantly associated with positive smoking status (P = 0.001), poorer tissue differentiation (P = 0.0052), higher TNM stage (P<0.0001), lymph node metastasis (P<0.0001), advanced tumor stage (P<0.0001), and poorer prognosis (P<0.0001). Multivariable analysis showed that FoxM1 expression increased the hazard of death (hazard ratio, 1.899; 95% CI, 1.016–3.551). Furthermore, by various *in vitro* and *in vivo* experiments, we showed that targeted knockdown of FoxM1 expression could inhibit the migratory and invasive abilities of NSCLC cells, whereas enforced expression of FoxM1 could increased the invasion and migration of NSCLC cells. Finally, we found that one of the cellular mechanisms by which FoxM1 promotes tumor metastasis is through inducing epithelial-mesenchymal transition (EMT) program.

**Conclusions:**

These results suggested that FoxM1 overexpression in tumor tissues is significantly associated with the poor prognosis of NSCLC patients through promoting tumor metastasis.

## Introduction

Lung cancer has remained the leading cause of cancer-related death worldwide for several years. The poor prognosis of lung cancer mainly due to little symptoms at early stage and when diagnosed, tumor cells have always metastasized to other organs [1]. Epithelial-mesenchymal transition (EMT) is substantial in tumor cell metastasis and characterized by the dissolution of intercellular junctions through the internalization and down-regulation of various proteins present in tight junctions, such as zonula occludens (ZO)-1, and adherens junctions, such as E-cadherin [2], [3].

Forkhead box M1 (FoxM1), a member of the Fox family of transcriptional factors, has been shown to be essential for cell cycle progression and is an important cell-cycle regulator controlling transition from G1 to S phase as well as entry into and completion of mitosis [4], . It has been reported to be over-expressed in a variety of tumors, including lung, liver and breast cancers [8], [9], [10], [11], [12], [13], and played an essential role in development and progression in various malignancies [11], [14], [15], [16]. Recently, FoxM1 was reported to drive hepatic fibrosis and metastasis in HCC [17], and It has been well documented that the hedgehog-signaling pathway was activated in the NSCLCs, and several molecules involved in this pathway, including PTCH1, SMO, GLI1, were observed to correlate with multiple prognostic parameters of the NSCLCs. Notably, these molecules were also observed to correlate with the increased expression of FoxM1 [18]. However, whether FoxM1 could exert a direct effect on the prognosis of NSCLCs patients remains to be elucidated. Yang and colleagues recently reported that FoxM1 overexpression correlated with the poor survival of patients using immunohistochemistry analyses of 69 cases of squamous cell carcinoma specimens [19]. However, the prognostic role of FoxM1 expression in other types of NSCLCs has not been determined. Liu et al. found that the expression status of FoxM1 in NSCLC is an independent prognostic factor and negatively correlated with the prognosis., but their study found no relationship between FoxM1 expression and other critical clinical parameters [20]. Meanwhile, all of the studies above did not demonstrate the potential underlying mechanisms. In our previous study, we have shown that FoxM1 promoted *in vitro* metastatic ability of small cell lung cancer [21]. In the current study, we aim to determine the prognostic roles of FoxM1 overexpression in NSCLCs and further explore the mechanism by which FoxM1 could contribute to the metastasis of NSCLCs.

## Results

### Patient Characteristics and Relationships between FoxM1 Expression and Clinicopathologic Characteristics

**Figure 1:**
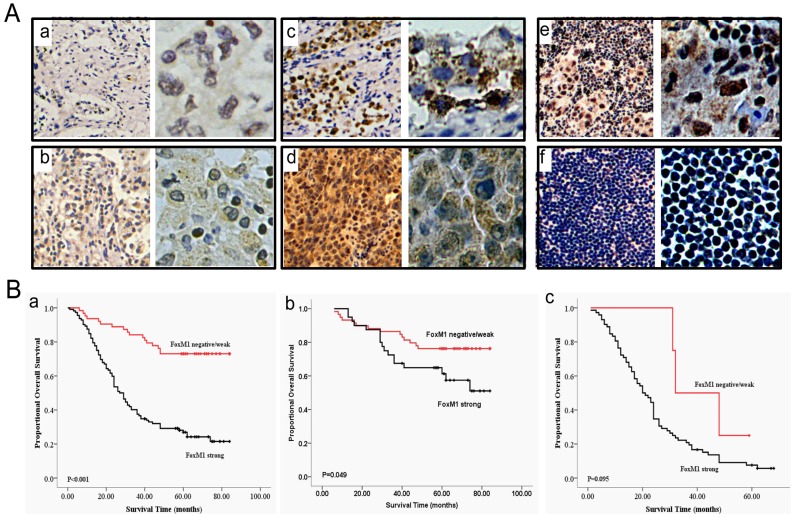
FoxM1 overexpression correlated with substantially poor prognosis of NSCLC patients. (A) Representative results of FoxM1 staining in NSCLC tumor tissues analyzed by immunohistochemistry (a, negative; b, weak; c, moderate; d, intense, respectively). Representative results of FoxM1 expression levels between lymph node positive (e) and lymph node negative (f) (a-f, left, magnification: ×100; right, magnification: ×400). (B) Overall survival analyses for all patients (a), patients with stage I/II (b) and patients with stage III/IV (c) according to the results of immunohistochemistry analysis. The log-rank test was used to test the two survival distributions. P<0.05 was considered to have statistical significance.

The clinicopathologic characteristics of the patients are summarized in Table S1. The overall follow-up durations ranged from 1 to 84 months (median, 42 months). To investigate whether the increased expression of FoxM1 was associated with various prognostic factors, patients were classified into two groups in terms of immunohistochemical staining for FoxM1 (Figure 1A, a–d): negative/weak expression and strong expression. As shown in Table S2, those patients with lymph node metastasis (Figure 1A, e) had a significantly higher expression of FoxM1 compared with those patients without lymph node metastasis (Figure 1A, f) (P<0.0001). Meanwhile, stronger FoxM1 expression was correlated with positive smoking status (P = 0.001), poorer differentiation (P = 0.0209), advanced tumor stage (P<0.0001) and higher TNM stages (P<0.0001), whereas no substantial difference was observed in patients’ age, gender and histology at the time of diagnosis between high and low levels of FoxM1.

### FoxM1 Expression Positively Correlated with Tumor Progression and Poor Survival of Non-small Cell Lung Cancer Patients

First, overall survival analysis was used to examine the correlation between FoxM1 expression and prognosis. The prognosis of those patients with a strong tumor expression of FoxM1 was significantly poorer than that of patients with a negative or weak tumor FoxM1 expression (P<0.0001) (Figure1B, a). In further analyses, FoxM1 expression was associated with poorer survival for patients with stage I/II cancer (P = 0.049) (Figure 1B, b) and for patients with stage III/IV cancer (P = 0.095) (Figure 1B, c), though the result of stage III/IV did not reach the significance. A univariate analysis showed that tumor stage (P<0.0001), lymph node status (P<0.0001), TNM stage (P<0.0001) and FoxM1 expression (P<0.0001), each predicted a significantly worse prognosis in NSCLC patients (Table 1). The clinical prognosis was, however, not associated with age, gender, smoking status, histology and differentiation.

**Table 1:** Univariate Survival Analysis (n = 175).

PARAMETER	HR	95% CI	P value	Median survival time (month)
Gender	1.173	0.776–1.773	0.4489	male: 47
				female: 41
Age	1.210	0.819–1.789	0.3384	≤55∶48
				>55∶36
Smoking status	1.478	0.989–2.208	0.057	No:62
				Yes:33
FoxM1expression	4.498	2.658–7.611	<0.0001*	weak: 48
				strong: 28
Histology	1.029	0.679–1.961	0.8917	AC: 41
				SCC: 47
Tumor stage	3.854	2.579–5.758	<0.0001*	T1, T2∶74
				T3, T4∶23.5
Lymph node metastasis	7.475	4.661–11.986	<0.0001*	No: 60
				Yes: 23
TNM Stage	6.625	4.249–10.329	<0.0001*	Stage I/II: 74
				Stage III/IV: 22.5
Differentiation	0.564	0.378–0.843	0.0052	Well and moderately differentiation: 31.5
				Poorly differentiation: 74

Abbreviation: No., number; TNM, tumor node metastasis; AC, adenocarcinoma; SCC, squamous cell carcinoma.

* significant.

Furthermore, a Cox proportional hazards model was applied to estimate the effect of FoxM1 expression on survival. The crude hazard ratio (HR) of FoxM1 strong expression compared with FoxM1 negative or weak expression was 1.899 (95% CI, 1.016–3.551, P<0.05), which indicated that strong FoxM1 status increased the hazard of lung cancer related death by nearly two times that of negative or weak FoxM1 status. With multivariable analysis, FoxM1 expression, lymph node metastasis and age were significantly associated with survival (Table 2). These findings suggest that FoxM1 seems to be an independent and significant predictor of poorer survival.

**Table 2:** Multivariable analysis for the effect of FoxM1 expression on survival.

Variable	Chi-Square	P value	Hazard ratio	95% CI
Age	4.497	0.034*	1.586	1.036–2.428
Gender	0.001	0.986	1.004	0.636–1.585
Smoking status	0.176	0.675	1.095	0.715–1.678
Histology	0.031	0.860	0.956	0.579–1.580
FoxM1 expression	4.033	0.045*	1.899	1.016–3.551
Tumor stage	1.163	0.281	1.345	0.785–2.304
Lymph nodemetastasis	6.454	0.011*	3.178	1.302–7.755
TNM Stage	1.144	0.285	1.628	0.667–3.975
Differentiation	0.001	0.974	1.008	0.636–1.597

Abbreviation: No., number; TNM, tumor node metastasis.

* significant.

### Targeted Knockdown of FoxM1 Expression Inhibited *in vitro* Metastatic Potentials of NSCLC Cells

**Figure 2:**
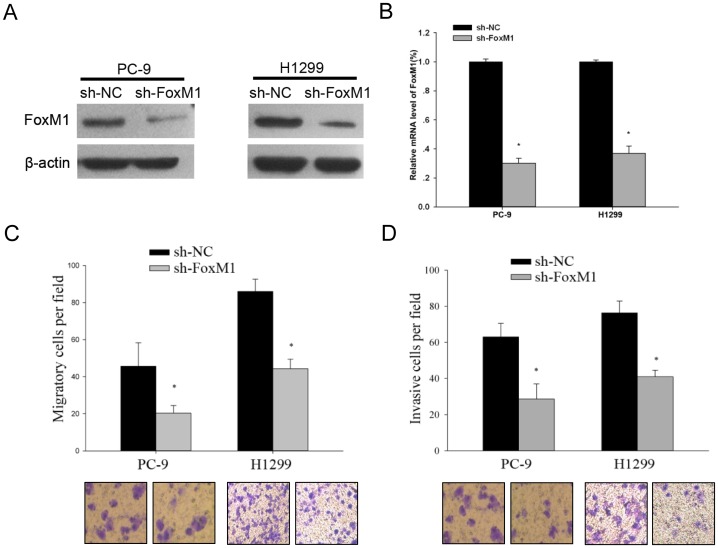
Targeted knockdown of FoxM1 expression inhibited the in vitro metastatic potentials of NSCLC cells. (A, B) The detection of lentivirus-mediated knockdown of FoxM1 in PC-9 and H1299 cells by WB and q-PCR, respectively. (C, D) A representative result of the trans-well migration assays for the effects of FoxM1 on the *in vitro* migratory and invasive abilities of PC-9 and H1299 cells. The results are shown as the mean ± s.d., *p<0.05.

The above results revealed that high FoxM1 expression was associated with poor prognosis of NSCLC patients, but the underlying mechanisms remain unclear. In our previous study, we have demonstrated that FoxM1 could mediate the proliferation of SCLC cell lines, which may be associated with the advanced tumor stage [21]. But the roles of FoxM1 expression in NSCLC cell migration and invasion are largely unknown. Thus, to find out whether FoxM1 mediates prognosis in lung cancer through promoting metastasis, we applied lenti-virus transduction to knock down the FoxM1 expression on the migratory and invasive ability of H1299 and PC-9 cells (Figure 2A and B). The results suggested that the disruption of FoxM1 expression could significantly inhibit the migratory and invasive abilities of NSCLC cells by trans-well migration assay (Figure 2C and D). Furthermore, we also found that in different two cell lines, knockdown of FoxM1 expression could significantly inhibit the proliferation rate at 48 and 72 hours (Figure S1A), and overexpression of FoxM1 promoted tumor proliferation after 72 hours in nude mice (Figure S1B), all of which were consistent with our previous results.

### Enforced Expression of FoxM1 Increased the *in vitro* and *in vivo* Metastatic Abilities of NSCLC Cells

**Figure 3:**
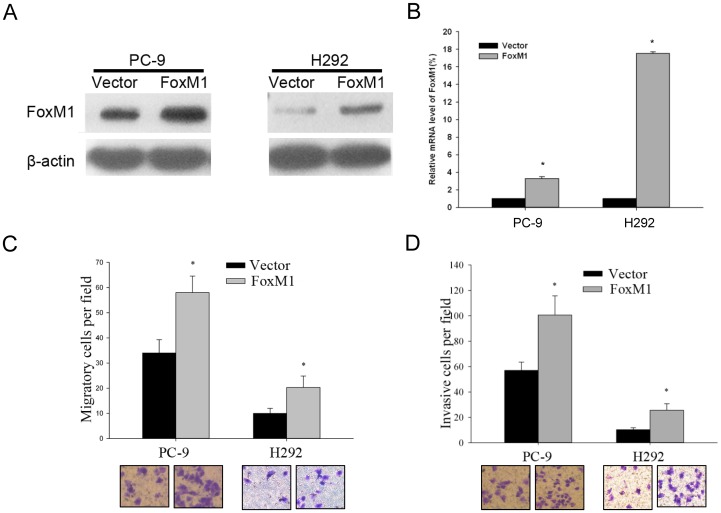
Ectopic expression of FoxM1 increased the in vitro metastatic potentials of NSCLC cells. (A, B) The detection of lentivirus-mediated overexpression of FoxM1 in PC-9 and H292 cells by WB and q-PCR, respectively. (C, D) A representative result of the trans-well migration assays for the effects of FoxM1 on the *in vitro* migratory and invasive abilities of PC-9 and H292 cells. The results are shown as the mean ± s.d., *p<0.05.

**Figure 4:**
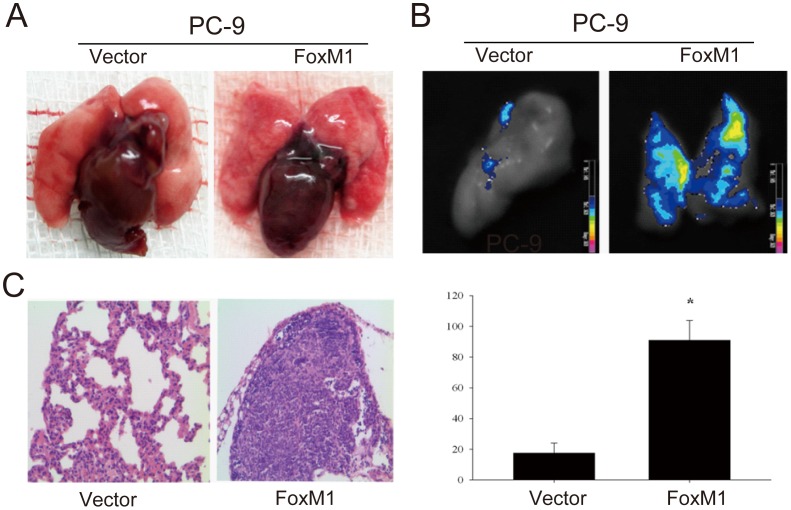
Enforced expression of FoxM1 promoted the in vivo metastatic abilities of NSCLC cells. (A, B) Visual inspection and fluorescence imaging analysis of lung metastatic nodules in two groups (PC-9-Vector and PC-9-FoxM1) of mouse models by tail vein injection of tumor cells, respectively. (C) The effects of *FoxM1* on the *in vivo* metastatic abilities of PC-9 cells in xenograft models of nude mice (n = 6) as determined by examination of mouse lungs for microscopic nodules. Representative results of histological examination of mouse lungs for metastatic nodules (left). The number of lung metastatic noudels in two groups of mouse models (right). The results are shown as the mean ± s.d., *p<0.05.

To further prove the role in NSCLC metastasis, we stably overexpressed FoxM1 expression in PC-9 and H292 cell lines (Figure 3A and B). First, by use of Trans-well assays, we showed that overexpression of FoxM1 could significantly increase the migratory and invasive potentials of NSCLC cells (Figure 3C and D). To further confirm the role of FoxM1 in the metastasis of non-small cell lung cancer, we developed a mouse model by tail vein injection of PC-9 FoxM1 overexpressing (PC-9-FoxM1) and control (PC-9-Vector) cells. Five weeks after cell inoculation, the mice were sacrificed to obtain the lungs (Figure 4A), and monitored by fluorescence imaging, the results of which showed that PC-9-FoxM1 cells displayed much stronger fluorescence signals in lungs than that of control cells (Figure 4B). Furthermore, histological study showed that the number of metastatic nodules in lungs of FoxM1 overexpression group was significantly higher than those in control group (Figure 4C and D). Taken together, these findings support the hypothesis that FoxM1 plays an important role in tumor metastasis of NSCLC.

### FoxM1 Induced the Epithelial-mesenchymal Transition of NSCLC Cells and Tumor Tissue Samples

**Figure 5:**
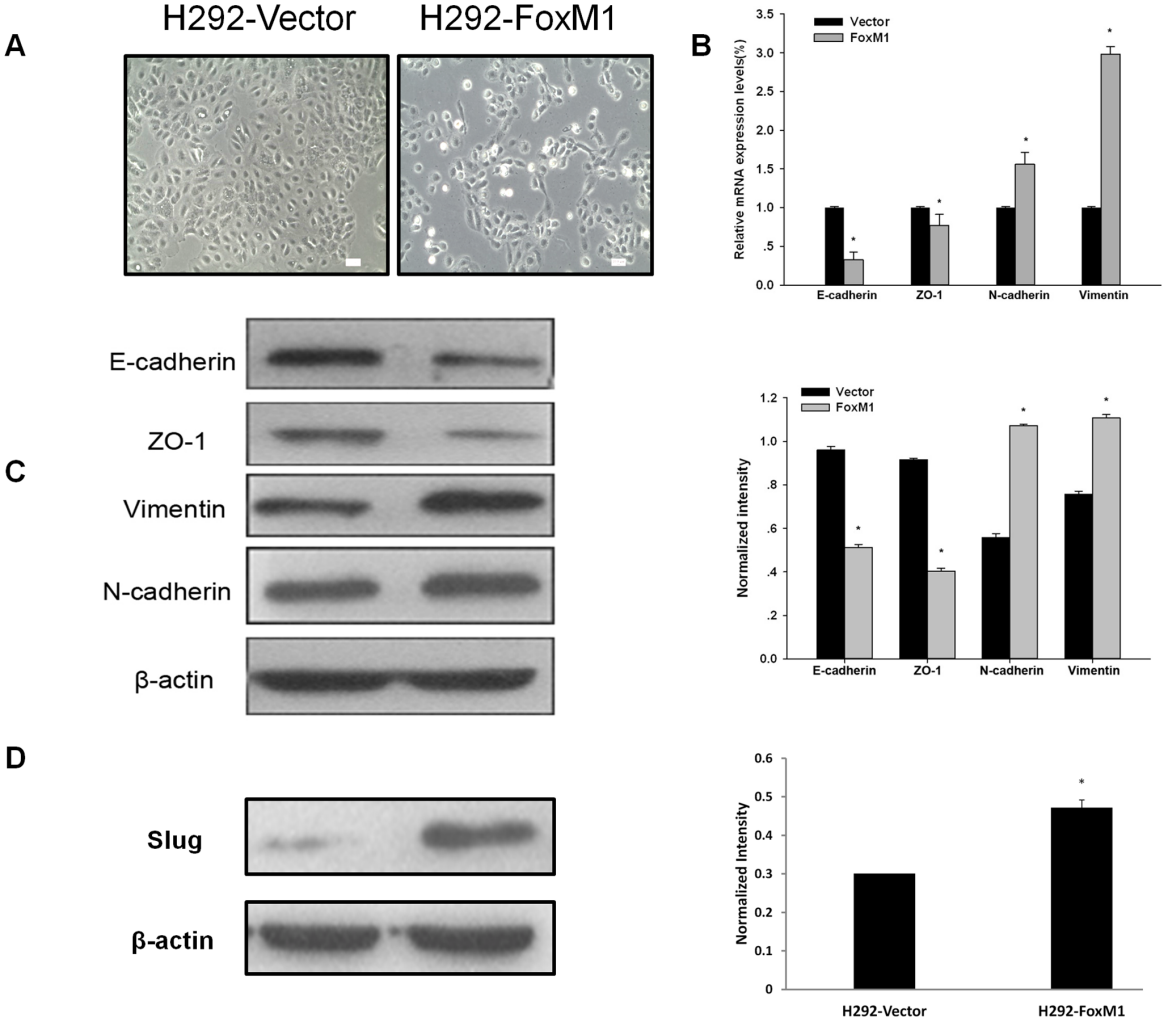
FoxM1 overexpression induced the epithelial-mesenchymal transition of NSCLC cells. (A) Representative phase-contrast images of the H292-vector and H292-FoxM1 cells (Magnification: 200×). (B) quantitive real-time PCR analysis of the relative expression of EMT markers as indicated. GADPH were used as loading control. Data are expressed as Mean ±s.d., *p<0.05. (C) The relative expression of EMT markers as indicated was determined by immunoblotting (left), and the results of which was further quantified (right). β-actin served as a loading control. (D) The relative expression of Slug as indicated was determined by immunoblotting (left), and the results of which was further quantified (right). β-actin served as a loading control.

We further observed cells with high FoxM1 expression, which were found to have phenotypic changes reminiscent of EMT. As shown in Figure 5A, distinct morphological differences between the H292-FoxM1 cell line and its parental H292-vector cell line were observed in cell culture. Whereas H292 cells are polygonal and grow in clusters, H292-FoxM1 cells are elongated and spindle-shaped, and grow in a scattering pattern to confluence. To further explore the mechanisms associated with the increased metastasis potential, we tested some essential factors associated with EMT, which promotes metastasis in carcinomas. We found that H292-FoxM1 cells expressed lower mRNA and protein levels of E-cadherin and ZO-1 while obtained higher mRNA and protein levels of the mesenchymal markers, like vimentin and N-cadherin (Figure 5B and C). Slug is a transcription repressor, which is potent inducer of EMT. We have observed increased Slug expression in FoxM1-overexpression cells (Figure 5D). Our observations suggest that FoxM1 induces metastasis through EMT.

**Figure 6:**
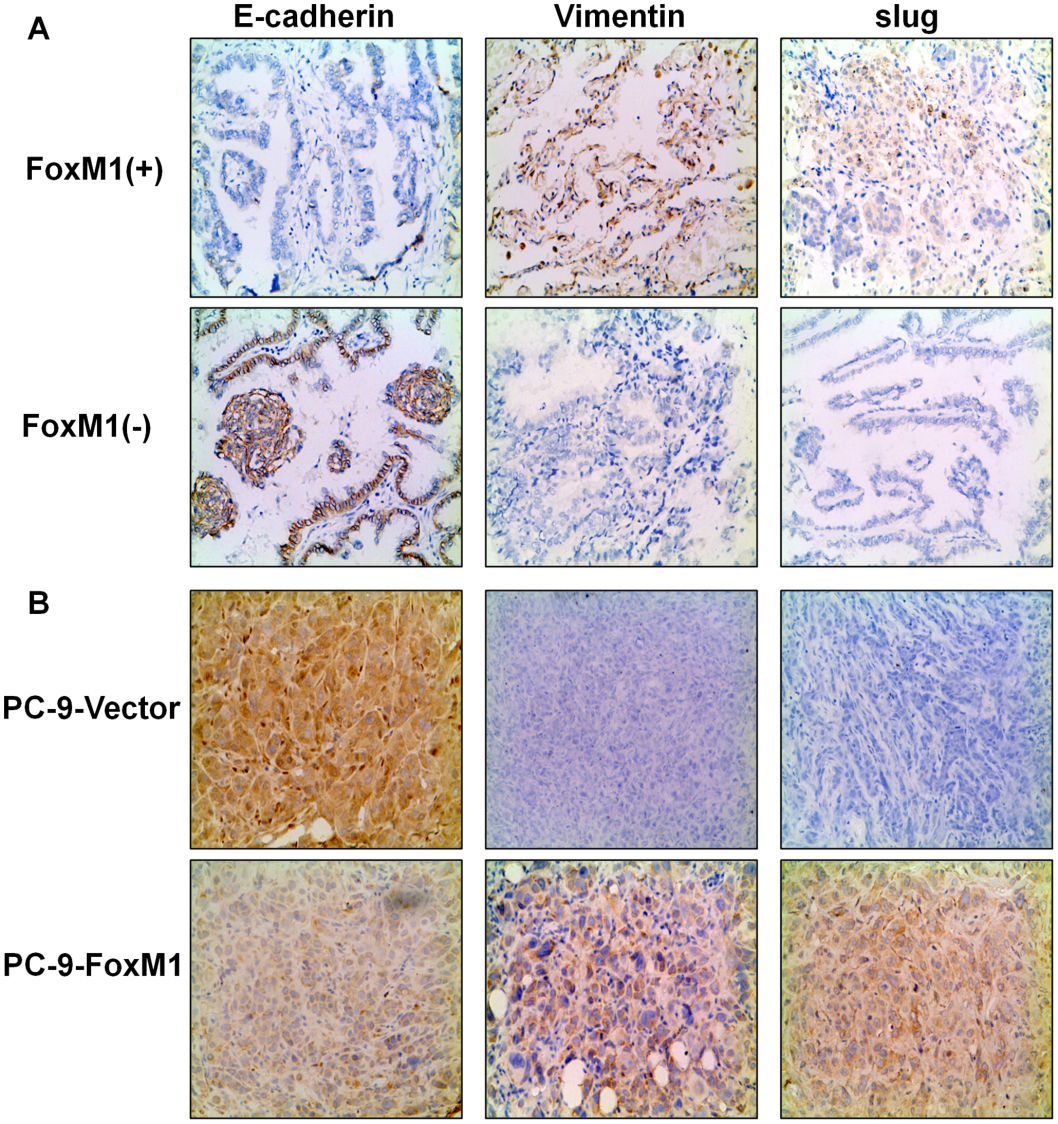
FoxM1 overexpression induced the epithelial-mesenchymal transition of NSCLC tissue samples. (A) The relative expression of E-cadherin, vimentin and Slug as indicated was determined by immunohistochemical staining in human tumor samples with high (above) and low (below) FoxM1 expression. (B) The relative expression of E-cadherin, vimentin and Slug as indicated was determined by immunohistochemical staining in nude mice tumors derived from PC-9-vector cells (above) and PC-9-FoxM1 cells (below).

To further demonstrate the relationship between FoxM1 expression and EMT markers, immunohistochemistry staining was carried out for E-cadherin, vimentin and Slug in tumors samples. In human lung cancer samples, increased vimentin and slug expression and decreased E-cadherin expression were found in the groups with high FoxM1 expression, while decreased vimentin and Slug expression and increased E-cadherin expression in the groups with low FoxM1 expression (Figure 6A). Furthermore, we have established subcutaneous solid tumor models. After 4 weeks of injection of PC-9-FoxM1 and PC-9-vector cells, solid tumors were harvested, embedded in paraffarin and stained for EMT markers. As shown in Figure 6B, compared with tumors derived from PC-9-vector cells, tumors derived from PC-9-FoxM1 cells revealed increased expression of Vimentin and Slug and decreased expression of E-cadherin (Figure 6B). Our observations in morphological and molecular changes suggest that FoxM1 overexpression induces an EMT-like phenotype through up-regulation of Slug in NSCLC cells.

## Discussion

In this study, we assessed the expression levels of FoxM1 using IHC staining in a cohort of 175 Chinese NSCLC patients and for first time demonstrated that elevated expression of FoxM1 was correlated with several clinicopathological factors and predicted an unfavorable prognosis in NSCLC patients, including SCC and AC. Moreover, we found that the deregulation of FoxM1 expression was significantly associated with higher metastatic abilities through inducing EMT.

Considering the role of FoxM1 in the tumor cell growth, some studies have evaluated the possibility of FoxM1 expression as a biomarker to predict the clinical outcome of NSCLC patients. Yang et al found that FoxM1 related with clinical factors and plays critical role in prognosis in 69 patients with SCC, not including AC. and Liu et al demonstrate that FoxM1 could be an independent factor for prognosis in 68 NSCLC patients, including 37 AC and 19 SCC, but the relationship between FoxM1 expression and other clinicoparameters was tested with no relationship. These paradoxical results may due to their different study population, small sample size and varied clinical data quality. In our study, we found that FoxM1 expression in tumor specimen was positively associated with several clinicopathological parameters, such as TNM stage, tumor stage and lymph node metastasis. Moreover, higher FoxM1 expression was correlated with a poor prognosis of NSCLC patients. These results indicated that FoxM1 was related with NSCLC progression and prognosis. However, underlying mechanisms needs to be further explored.

Metastasis involves a series of complex steps [22], including decreased adhesion, increased motility, cell attachment, matrix dissolution, and migration [23]. During tumor progression, cancer cells undergo dramatic changes in cytoskeletal organization to adopt invasive phenotype and eventually metastasize to other organs. EMT is a key process in tumor metastasis [24], [25], [26], which associated with reduced cell-surface expression of E-cadherin, increased N-cadherin [27], [28] and altered expression of several cyto-skeletal proteins, playing a significant role towards the migratory activity of the tumor cells [29].

Our data revealed that inhibition of FoxM1 could suppress the metastatic ability, while up-regulated expression level of FoxM1 in lung cancer cells enhanced the migratory and invasive properties in vitro and in vivo. Levels of E-cadherin, ZO-1 were elevated and vimentin, N-cadherin were reduced with higher FoxM1 expression. Also, FoxM1 regulated Slug expression, which is the inducer of EMT. These results suggested that FoxM1 was associated with prognosis by promoting metastasis of tumors cells through inducing EMT. Hyun JP et al. also demonstrated that in HCC, FoxM1 could activate the Akt-Snail1 pathway and involve in EMT transition [17]. Moreover, there are some other studies indicated that MMPs have emerged as central factors in both local growth and distant metastasis in tumors [30], [31], and FoxM1 could also regulate MMPs to promote OSCLC metastasis. Taken together, FoxM1 may play several significant functions in tumor cells. Its complete role in pathway concerning metastasis worths further examination.

In summary, our findings suggest that FoxM1 up-regulation may have effect on NSCLC prognosis and progression. We showed that FoxM1 overexpression was related to reduced overall survival and rapid tumor progression in NSCLC patients. Moreover, overexpression of FoxM1 could increase the migratory and invasive abilities of NSCLC cells. These results suggest that the FoxM1 expression is critical for the invasiveness of malignant NSCLC cells, and this effect was at least, in part through epithelial- mechenmychal transition. Therefore, our observation makes FoxM1 an attractive target of lung cancer therapy, and could be used as a biomarker for predicting prognosis.

## Materials and Methods

### Ethics Statement

Human participants involved in this research, provided their written informed consent according to the principles expressed in the Declaration of Helsinki. Medical Ethics and Human Clinical Trial Committee at Zhongshan Hospital, Fudan University has approved this study as well as the consent procedure (Permit Number: 2011–221). All animal protocols were approved by the Ethical Committee on Animal Experiments of the University of Fudan Animal Care Committee, Shanghai, China(Permit Number: SYXK:2008–0039). All efforts were made to minimize suffering.

### Patients and Tumor Samples

A total of 232 histologically confirmed NSCLC patients were consecutively recruited between January 2005 and February 2008 for the treatment of non-small cell lung cancer (NSCLC) at Zhongshan Hospital, Fudan University. Four patients were excluded if they previously received radiotherapy and/or chemotherapy. Chest X-ray and computed tomography were conducted for all the patients before operation. There are 142 patients underwent curative surgical resection. Chemotherapy with a cisplatin-based regime was applied for most patients at high stage (including IIIB and IV) or with high risk of recurrence and metastasis according to the treatment guideline for NSCLC at the surgical time. For this study, 53 patients were excluded from analysis, including 38 patients with poor quality and/or quantity of tissue samples, 11 patients with incomplete clinical data and 4 patients died of other causes originally were excluded from our analysis. Finally, a total of 175 patients were included in this study (Table S1). A total of 122 men and 53 women were included, including 97 smokers and 78 non-smokers, with ages ranging from 30 to 77 years, of which 89 were less than 55 years old. There were 76 stage I, 23 stage II, 51 stage III and 25 stage IV diseases, including 91 patients with no nodal metastasis and 84patients with lymph node metastasis. There were 36 patients with tumor of stage 1, 75 with stage 2, 25 with stage 3 and 39 with stage 4 and 118 patients with adenocarcinoma (AC), while 57 with squamous cell carcinoma (SCC). In terms of differentiation, there were 93 patients with well and moderately differentiation and 82 with poorly differentiation. A total of 74 patients were alive at the end of the follow-up, 101 patients died of lung cancer, and patients died of other causes or lost to follow-up were excluded from the study.

Clinicopathological information for each patient, including gender, age, smoking status, tumor stage, nodal status, TNM stage, histological grade and overall survival, was obtained retrospectively from clinical records and pathological reports. Survival time was defined as the duration from the date of diagnosis to the date of death or the end of the follow-up. The TNM status was determined according to the 7th edition staging system for NSCLC [32].

### Immunohischemistry Assay

An antibody against FoxM1 (Sigma Aldrich Inc., MO, USA) and a standard immunohistochemical technique were used for detecting FoxM1 expression as previously described [19], [20]. FoxM1 was observed in cytoplasmic or cytoplasmic and nuclear of the cells. Staining was assessed in five high-powered fields at 200×magnification. The staining score was categorized into four groups as negative = 0, weak = 1, moderate = 2 and intense = 3 [20]. The percentage area stained positive was categorized into four groups: less than 25% tumor cells positive = 0; 25% to 50% tumor cells positive = 1; 50% to 75% tumor cells positive = 2; more than 75% tumor cells positive = 3. Labeling score was determined by multiplying intensity score by the percentage area stained positive, which scores as 0, 1, 2, 3, 4, 6 and 9.The staining score was categorized into two groups as weak/negative staining (score <4) and strong staining (score ≥4). This threshold was determined by visually determining a clear positive stain supported by histograms of the range of scores [20], [33], [34]. The highest labeling score among the three tissue sections was entered for statistical analyses. The pathologists who performed the immunohistochemical assessment of FoxM1 were blinded to the patients’ histopathologic and follow-up data.

### Epidemiologic and Clinical Data Collection

Patients’ demographic parameters and smoking history were obtained through in-person interview at the time of initial visit, follow-up in the clinics or medical chart review. An individual who smoked more than 100 cigarettes in history was defined as an ever smoker, otherwise as a never smoker [35]. Follow-up information on patient death and survival was updated at 3-month intervals through onsite interview, direct calling, or medical chart review. The latest follow-up in this study was carried out on May 2012.

### Cell Lines, Cell Culture and Chemotherapeutic Reagents

The human lung adenocarcinoma cell line PC-9 and human lung cancer cell line NCI-H292, NCI-H1299 were all obtained from Cellular Institute of Chinese Academy of Science (Shanghai, China) [36]. These cells were cultured at 37°C under a 5% CO2 atmosphere in Dulbecco's Modified Eagle's Medium (DMEM) supplemented with 10% fetal bovine serum (FBS, Hyclone Inc., UT, USA), 100 U/ml penicillin, and 100 µg/ml streptomycin. Cells were regularly certified as free of Mycoplasma contamination.

### shRNA Experiments

The lenti-virus shRNA vector was constructed as described previously [37]. Briefly, FoxM1 and negative control shRNA were subcloned into the MluI/ClaI sites of a pLVTHM vector (Addgene) with the following oligonucleotides respectively:5′-CGCGTCGGCCGGAACATGACCATC AATTCAAGAGATTGATGGTCATGTTCCGGCTTTTTTCCAT-3′and 5′-CGATGGAAAAAAGCCGGAACATGACCATCAATCTCTTGAATTGATGGTCATGTTCCGGCCGA-3′for FoxM1, and 5′-CGCGTCGGT AGCGACTAAACACATCAATTCAAGAGATTGATGTGTTTAGTCGCTACTTTTTTCCAT-3′and 5′-CGATGGAAAAAAGTAGCGACTAAAC ACATCAATCTCTTGAATTGATGTGTTTAGTCGCTACCGA-3′for the negative control. Lenti-virus generation and infection of H1299 and PC-9 cells were performed as described above.

### Transduction of Tumor Cells

Plasmid (EX-Z5438-LV135) and Lenti-Pac™ HIV Expression Packaging Kit were purchased from Gene Copoeia Inc.(Gene Copoeia Inc.,Guangzhou, China) Transductions of H292 and PC-9 cells were performed according to instructions supplied by the manufactures. Stable transfectants were further confirmed by RT-PCR and immunoblotting on the basis of their FoxM1 expression.

### Tumor Cell Migration and Invasion Assays

Assays to measure tumor cell migration were performed in a modified Boyden chamber (Transwell, Corning Costar, MA, USA) containing a gelatin-coated polycarbonate membrane filter (8 µm pore size). Bio-Coat Matrigel (BD Biosciences, Bedford, MA, USA), which reconstitutes basal membrane, was used to assess cell invasion. The degree of tumor cell migration and invasion was evaluated according to previous protocols [38]. Non-migrated cells were removed from the upper side of the membrane by scrubbing. Cell counting was accomplished by Coomassie blue staining and cells were visualized under a microscope (Leica Inc., Solms, Germany) subsequently.

### Animal Experiments

Four- to five-week-old male BALB/CA nude mice (purchased from Shanghai Institute of Material Medicine, Chinese Academy of Science, Shanghai, China ) were maintained under specific pathogen-free (SPF) conditions. The method used is same as the one described previously for generating breast cancer model [39]. PC-9-FoxM1 cells (2 × 10^6^ per mouse) stably expressing FoxM1 or vector were injected into the tail vein of nude mice (n = 6). Five weeks later, the mice were sacrificed and the lungs were removed. After that, these lungs were processed for histological examination and monitored for potential lung tumor metastases by fluorescence imaging [40]. Metastatic lesions were identified by fluorescence signals (Night Owl LB 981 *In Vivo* Imaging System, Berthold, Germany) and analyzed by WinLight32 software. For histological analysis, the lungs were harvested at necropsy and fixed in 10% formalin. The fixed samples were then embedded in paraffin, and three non-sequential serial sections were obtained per animal. The sections were stained with H&E and analyzed for the presence of metastasis.

### Statistical Analysis

Several clinicopathological factors were evaluated. Fisher’s exact test was used to evaluate the correlation between the clinicopathological variables and the expression of FoxM1. The clinicopathological variables and the expression of FoxM1 were taken into account for the analysis of survival on the basis of the Kaplan–Meier method; Multivariable analysis was performed with Cox proportional hazards regression model to examine the independent prognostic effect of FoxM1 on survival by adjusting for confounding factors. A P-value<0.05 was considered to be significant in all analyses. SPSS 17.0 was used to do the statistical analyses in this paper.

## Supporting Information

Figure S1 Effects of altered FoxM1 expression on proliferation rate *in vitro* and *in vivo*. (A) Proliferation rate of FoxM1 of SPC-A-1 sh-FoxM1(left) and H1299 sh-FoxM1 (right) cells. FoxM1 expression was first stably knocked-down by lenti-virus infection in SPC-A-1 and H1299 cells. Proliferation rates of cells in the groups transfected with shRNAs against FoxM1 (sh-FoxM1) (red line) decreased significantly, as compared with the shRNA-targeting negative control (sh-NC) (blue line). Data are expressed as mean±SD. (B) Proliferation rate of tumors derived from PC-9-FoxM1cells in vivo. PC-9-vector and PC-9-FoxM1 cells were subcutaneously injected into nude mice. The tumor diameter was measured at the indicated time points. Proliferation rate of tumors derived from PC-9-FoxM1 cells (blue line) increased significantly, as compared with that from PC-9-vector cells (red line). Data are expressed as mean±SD.
(JPG)

Table S1 Characteristics of patients with non-small cell lung cancer.
(DOCX)

Table S2 Clinical profile and correlation between the clinicopathological features and expression of FoxM1.
(DOCX)
