# Supplementary figures and images for: FoxM1 Is Associated with Poor Prognosis of Non-Small Cell Lung Cancer Patients through Promoting Tumor Metastasis
Source: PLoS One. 2013 Mar 25;8(3):e59412. doi: 10.1371/journal.pone.0059412 (PMC3607616; doi:10.1371/journal.pone.0059412)

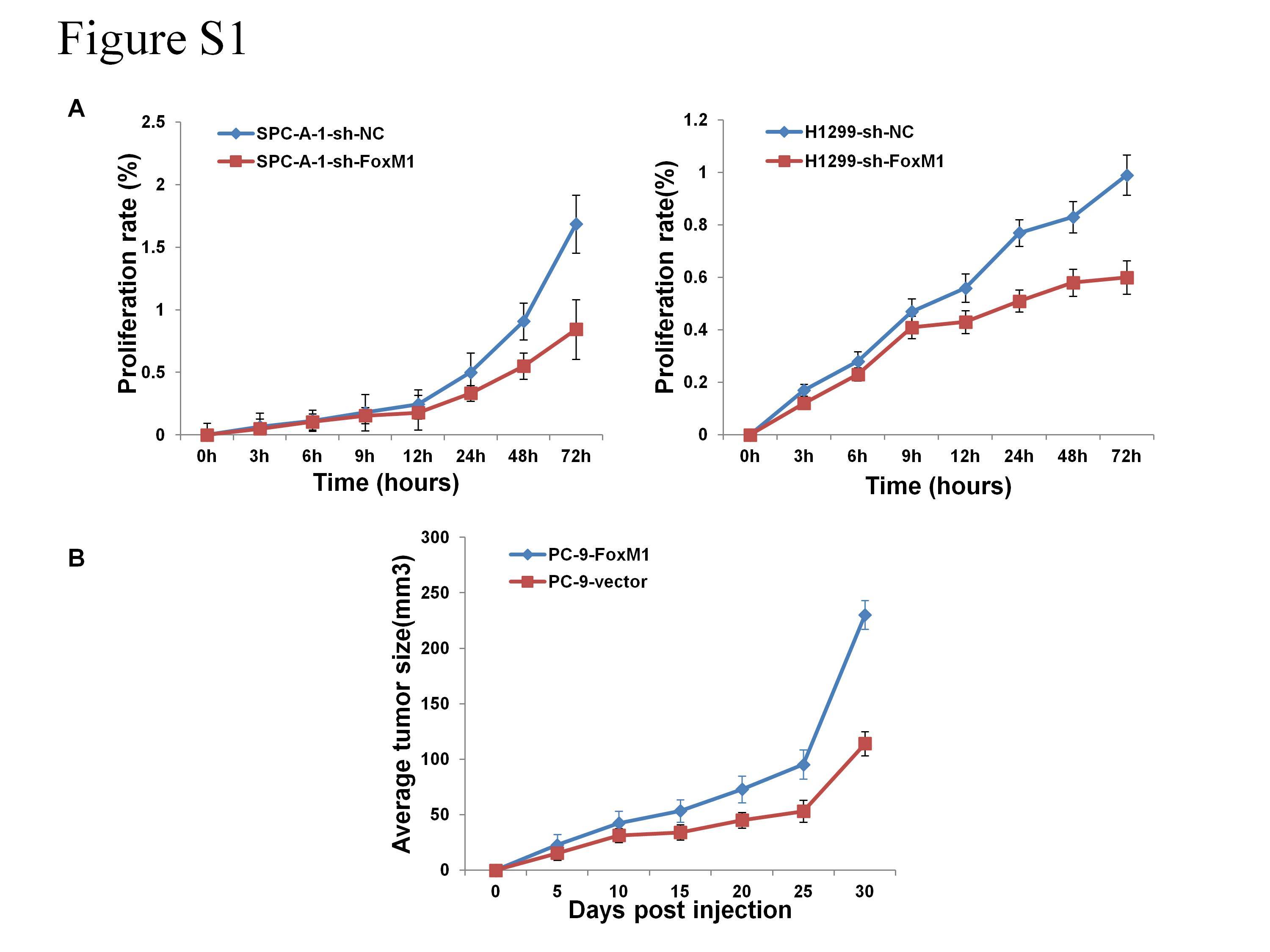

Supplement: Figure S1 — Effects of altered FoxM1 expression on proliferation rate in vitro and in vivo. (A) Proliferation rate of FoxM1 of SPC-A-1 sh-FoxM1(left) and H1299 sh-FoxM1 (right) cells. FoxM1 expression was first stably knocked-down by lenti-virus infection in SPC-A-1 and H1299 cells. Proliferation rates of cells in the groups transfected with shRNAs against FoxM1 (sh-FoxM1) (red line) decreased significantly, as compared with the shRNA-targeting negative control (sh-NC) (blue line). Data are expressed as mean±SD. (B) Proliferation rate of tumors derived from PC-9-FoxM1cells in vivo. PC-9-vector and PC-9-FoxM1 cells were subcutaneously injected into nude mice. The tumor diameter was measured at the indicated time points. Proliferation rate of tumors derived from PC-9-FoxM1 cells (blue line) increased significantly, as compared with that from PC-9-vector cells (red line). Data are expressed as mean±SD. (JPG) [file pone.0059412.s001.jpg]
