# Supplementary material for: FoxM1 Is Associated with Poor Prognosis of Non-Small Cell Lung Cancer Patients through Promoting Tumor Metastasis
Source: PLoS One. 2013 Mar 25;8(3):e59412. doi: 10.1371/journal.pone.0059412 (PMC3607616; doi:10.1371/journal.pone.0059412)
Supplement: Table S1 — Characteristics of patients with non-small cell lung cancer. (DOCX) [file pone.0059412.s002.docx]

**Supplementary tables**

**Table S1:** *Characteristics of patients with non-small cell lung cancer*

| *Characteristics* | *Patients(No.=175)* |
| --- | --- |
| *Age (years)* |  |
| ≤55 | 89(50.9%) |
| >55 | 86(49.1%) |
| *Gender* |  |
| Male | 122(69.7%) |
| Female | 53(30.3%) |
| *Smoking status* |  |
| Yes | 97(55.4%) |
| No | 78(44.6%) |
| *Histology* |  |
| Adenocarcinoma | 118(67.4%) |
| Squamous cell carcinoma | 57(32.6%) |
| *Differentiation* |  |
| Well and moderately | 93(53.1%) |
| Poorly | 82(46.9%) |
| *TNM Stage* |  |
| I | 76(43.4%) |
| II | 23(13.2%) |
| III | 51(29.1%) |
| IV | 25(14.3%) |
| *Tumor stage* |  |
| T1 | 36(20.6%) |
| T2 | 75(42.8%) |
| T3 | 25(14.3%) |
| T4 | 39(22.3%) |
| *Lymph node metastasis* |  |
| No | 91(52%) |
| Yes | 84(48%) |

Abbreviation: No., number; TNM, tumor node metastasis.

*, significant.
