# Supplementary material for: FoxM1 Is Associated with Poor Prognosis of Non-Small Cell Lung Cancer Patients through Promoting Tumor Metastasis
Source: PLoS One. 2013 Mar 25;8(3):e59412. doi: 10.1371/journal.pone.0059412 (PMC3607616; doi:10.1371/journal.pone.0059412)
Supplement: Table S2 — Clinical profile and correlation between the clinicopathological features and expression of FoxM1. (DOCX) [file pone.0059412.s003.docx]

**Table S2:** *Clinical profile and correlation between the clinicopathological features and expression of FoxM1.*

| *Variables* | *No. of patients* | *FoxM1 expression* | | *P-value* |
| --- | --- | --- | --- | --- |
|  |  | *Negative/Weak* | *Strong* |  |
| *Age(years)* |  |  |  | 0.5205 |
| ≤55 | 89 | 30 | 59 |  |
| >55 | 86 | 33 | 53 |  |
| *Gender* |  |  |  | 0.2911 |
| Male | 122 | 47 | 75 |  |
| Female | 53 | 16 | 37 |  |
| *Smoking status* |  |  |  | 0.001* |
| Yes | 97 | 28 | 69 |  |
| No | 78 | 35 | 43 |  |
| *Histology* |  |  |  | 0.6189 |
| Adenocarcinoma | 118 | 41 | 67 |  |
| Squamous cell carcinoma | 57 | 22 | 35 |  |
| *Differentiation* |  |  |  | 0.0209* |
| Well and moderately | 93 | 27 | 66 |  |
| Poorly | 82 | 37 | 45 |  |
| *TNM Stage* |  |  |  | <0.0001* |
| I, II | 99 | 59 | 40 |  |
| III, IV | 76 | 4 | 72 |  |
| *Tumor stage* |  |  |  | <0.0001* |
| T1 and T2 | 111 | 57 | 54 |  |
| T3 and T4 | 64 | 6 | 58 |  |
| *Lymph node metastasis* |  |  |  | <0.0001* |
| No | 91 | 57 | 34 |  |
| Yes | 84 | 6 | 78 |  |

Abbreviation: No., number; TNM, tumor node metastasis.

*, significant.
